# Is telemedicine a holy grail in healthcare policy: clinicians’ and patients’ perspectives from an Apex Institution in Western India

**DOI:** 10.1186/s12913-022-09013-y

**Published:** 2023-02-15

**Authors:** Nainsi Gupta, Manoj Kumar Gupta, Nitin Kumar Joshi, Neha Mantri, G. Sridevi, Mamta Patel, Akhil Dhanesh Goel, Kuldeep Singh, M. K. Garg, Pankaj Bhardwaj

**Affiliations:** 1 School of Public Health, All India Institute of Medical Sciences Jodhpur, Jodhpur, Rajasthan India; 2 Department of Community Medicine & Family Medicine, All India Institute of Medical Sciences Jodhpur, Jodhpur, Rajasthan India; 3 Department of Pediatrics, All India Institute of Medical Sciences Jodhpur, Jodhpur, Rajasthan India; 4 Department of Medicine, All India Institute of Medical Sciences Jodhpur, Jodhpur, Rajasthan India

**Keywords:** Telemedicine, Teleconsultation, COVID-19, Perception, Patient satisfaction

## Abstract

**Background:**

This study aimed to explore the perception of doctors regarding telemedicine consultations and the level of patient satisfaction with the services received through teleconsultations.

**Methods:**

This cross-sectional study was conducted on clinicians who provided teleconsultations and patients who received teleconsultations in an Apex healthcare institution in Western India. Semi-structured interview schedules were used to record the quantitative and qualitative information. Clinicians’ perceptions and patients’ satisfaction were assessed using two different 5-point Likert scales. Data were analyzed using SPSS v.23 using non-parametric tests (Kruskal Wallis and Mann-Whitney U).

**Results:**

A total of 52 clinicians who delivered teleconsultations and 134 patients who received teleconsultations from those doctors were interviewed in this study. For 69% of doctors, telemedicine was feasible to implement, and for the rest, it was challenging. Doctors believe telemedicine is convenient for patients (77%) and prevents the transmission of infection (94.2%). Difficulty in clinical evaluation (73%), communication (55.7%), network connectivity (34%), diagnosis and investigations (32%), and patients’ e-illiteracy (32%) were the most common challenges faced by clinicians. Patients’ experiences were positive in terms of ease of registration (82.1%), audio quality (100%), freedom to discuss medicine (94.8%), and comprehension of the diagnoses (88.1%). Patients expressed satisfaction with the length of the teleconsultation (81.4%), the advice and care they received (78.4%), and the manner and communication of the clinicians (78.4%).

**Conclusions:**

Though there were some challenges in the implementation of telemedicine, the clinicians perceived it quite helpful. The majority of the patients were satisfied with teleconsultation services. Difficulty in registration, lack of communication, and a deep-rooted mindset of physical consultations were the primary concerns from the patient side.

**Supplementary Information:**

The online version contains supplementary material available at 10.1186/s12913-022-09013-y.

## Background

Telemedicine ensures the high-quality capability to enhance the management of health care and distribution of health services in an accessible, efficient, peculiar, and cost-effective manner mitigating the shortage of health professionals [1]. By allowing patients to attend consultations remotely, telemedicine helps to prevent the spread of infection and exposure to a variety of illnesses by minimizing the need for unnecessary in-person visits and hospital overcrowding [2]. Patients may benefit from telemedicine by being more knowledgeable and engaged in their treatment plans and overall health awareness. Additionally, it offers a reliable platform for patients to quickly and conveniently get specialist consultations, e-prescriptions, and other health services [3].

Social distancing and frequent lockdowns during the COVID-19 pandemic had a cumulative effect on the diagnosis and treatment of diseases, enhanced the comorbidity issues among people, and caused unprecedented challenges to healthcare systems worldwide. Since the beginning of the pandemic, many people in need of treatment could not get the health services and medications they require. As a result, most countries implemented telemedicine as an effective and safer tool for health service delivery.

To continue healthcare services during the COVID-19 pandemic, like many other countries, India responded by implementing telemedicine and other digital health innovations. On March 25th, 2020, the Ministry of Health and Family Welfare (MoH&FW), Government of India (GoI), released the ‘Telemedicine Practice Guidelines’ for Registered Medical Practitioners (RMPs) [4]. Several healthcare establishments began offering teleconsultations within a week of the closure of hospital OPDs in India.

Although this guideline is expected to bring transformation in health care delivery, it might have faced some challenges during the actual implementation in health facilities. Some healthcare professionals and patients resist acquiring telemedicine services because of traditional thoughts and the dearth of technological literacy & skills to implement telemedicine [5].

There is a need to explore the facilitating factors, barriers/challenges, and satisfaction levels of patients from different geographical regions so that this evidence may be helpful in the improvement of telemedicine implementation in the future. So, this study was planned to assess the facilitators and barriers to implementing telemedicine services. Further, the perception of the doctors regarding telemedicine consultations and the level of patient satisfaction with the services received through teleconsultation was also assessed.

## Methods

This cross-sectional study was conducted in an apex healthcare institution in western India from May 1st to July 31st, 2021. The study included clinicians who provided teleconsultation services and patients who received teleconsultations from the hospital’s Out Patient Departments (OPDs).

The telemedicine set-ups were started in OPDs of 27 different clinical departments. Dedicated computers and large display units/TVs with 24 × 7 high-speed internet connectivity were provided in all the set-ups. The arrangements were made in the quiet rooms to avoid the background noise. Rosters for clinicians were prepared to manage the teleconsultations in OPDs. The facility was started for patients who were already registered with the apex healthcare institution and were in consultation or under treatment with any department. It was declared that this facility was not a substitute for any medical emergency. Dedicated helpdesk numbers were made publically available as the first point of contact and patients were connected to the respective department through those helpdesks which were operating on working days. Besides that, a department-wise list of phone numbers was also publically displayed so that patients can also directly book their appointment in the respective department. It was declared that when a patient calls/contacts for telephonic consultation, it is implied that the patient has agreed to telephonic consultation for guidance regarding their medical issues; to share important and especially private information about their medical problems, and agree to be provided consultation by any doctor of the department contacted. Calling patients also agree and accept the prescriptions through telephone / what’s app etc.

This was a time-bound study. Two consultants/doctors from 27 different clinical departments were interviewed. Thus, a total of 54 doctors (27 × 2) were interviewed physically (face to face). Only those consultants who were active in offering telemedicine services were chosen on purpose.

Lists of patients who had undergone teleconsultation in the previous month were obtained from each department’s relevant authority. These patients were already registered with the apex healthcare institution and directly booked their appointments online by themselves or with the help of the caregiver. Patients either used personal mobile phones or laptops to receive the teleconsultations. Five patients from each department were chosen from the lists by simple random sampling. For all the patients (including pediatric patients), the main person who received the teleconsultation (patient or attendant) was interviewed. A total of 134 patients were interviewed in this study.

A female investigator pursuing an MPH (Master in Public Health), who was already trained in conducting qualitative research, took all the interviews. The interview of clinicians was conducted in the healthcare institution itself. The patients were interviewed over the phone. Each interview (both for clinicians and patients) lasted for approximately 25 minutes. Privacy was maintained during the interviews. Both quantitative and qualitative data were collected through specifically designed different semi-structured interview schedules and interview guides for doctors and patients, which were developed based on the literature review of comparable studies [6, 7]. Audio-video recordings of the interviews could not be done due to denial by a majority of the participants.

Two separate five-point Likert scales were designed to assess doctors’ and patients’ perceptions of telemedicine services. The scales were rated as 5 for Strongly Agree (SA), 4 for Agree (A), 3 for Not Sure (NS), 2 for Disagree (D), and 1 for Strongly Disagree (SD). Domains such as knowledge, experience, satisfaction, necessity, strengths, and weaknesses of telemedicine services were included in the tool intended for doctors. For patients, the domains dealt with their experience in telemedicine services, satisfaction level, and attitude toward teleconsultation. Pilot testing of the tools was done on five doctors and ten patients not included in the final list sampling.

The study was prior approved by the Institutional Ethics Committee. Informed written consent was taken from the doctors, while only verbal consent was obtained from the patients before conducting the interview. Data were analyzed using SPSS v.23. Inferences were drawn using descriptive statistics and non-parametric (Kruskal Wallis and Mann-Whitney U) tests.

## Results

A total of 52 clinicians who delivered teleconsultations and 134 patients who received teleconsultations from those doctors were interviewed in this study. 61.5% of the doctors were in the age group 30-40 years, and 78.8% were male. Doctors were divided into two groups: Faculties and Senior Residents (SRs), with the latter accounting for 61.5%. The majority (85.1%) of the patients were in the 15-59 years age group; 9.7% were in the pediatric age group (≤14 years), and 5% were in the elderly (≥60 yrs) age group. The mean age of the patients was 35.33 ± 15 years. The number of male patients (58.2%) was higher than female patients (41.8%). Most patients (77.4%) had completed at least secondary education (10th standard). Among the patients, 59.1% were unemployed, including students and homemakers (Table 1).

**Table 1:** Socio-demographic attributes of Doctors and Patients

Variables	No. (%)
**Doctors (** ***N*** **= 52)**	
**Age**	
< 30 yrs	16 (30.8)
30-40 yrs	32 (61.5)
> 40 yrs	4 (7.7)
**Gender**	
Male	41 (78.8)
Female	11 (21.2)
**Occupational Groups**	
Faculties	20 (38.4)
Senior resident	32 (61.5)
**Patients (** ***N*** **= 134)**	
**Age**	
Pediatrics (≤14 yrs.)	13 (9.7)
Adult (15-59 yrs)	114 (85.1)
Elderly (≥60 yrs)	7 (52.0)
**Gender**	
Male	78 (58.2)
Female	56 (41.8)
**Education** ^a^ **(** ***n*** **= 120)**	
Illiterate	2 (1.6)
Just literate (1st to 4th standard)	3 (2.5)
Primary (5th to 7th standard)	15 (12.5)
Middle (8th to 9th standard)	7 (5.8)
Secondary (10th to 11th standard)	17 (14.1)
Intermediate(12th)	24 (20.0)
Graduate & Postgraduate	52 (43.3)
**Occupation** ^a^ **(** ***n*** ** = 120)**	
Agriculture & Animal work, Milkman	1 (0.8)
Job (Government / private/ business)	44 (36.6)
Labour (Skilled & Unskilled)	4 (3.3)
Unemployed/Students/Homemakers)	71 (59.1)

^a^Data not applicable for 14 Pediatric patients

As much as 69% of the doctors expressed that telemedicine was feasible to implement. 21% felt it was challenging to implement, and 10% thought it was difficult. On exploring the challenges faced by clinicians, the majority (73%) felt that clinical evaluation of patients through telemedicine poses a significant challenge. Other difficulties included problems with linguistics and communication (55.7%), poor network connectivity (34%), issues of diagnosis and investigations (32%), patients’ e-illiteracy (32%), patients’ absenteeism during follow-ups (16%), and problems with medicine prescription (2%).

To overcome those challenges, about 27% of the doctors called patients for an in-person visit for a clinical examination. While four doctors managed to mobilize technical (additional audio-visual) resources to ensure smooth communication, 19.2% were able to resolve the issue simply by calling the patients over the phone. Other methods for overcoming communication barriers included requesting video recordings and photographs, asking someone else to communicate, and having patients write down their problems and transmit them via email and WhatsApp. Doctors also encouraged patients to share the results after conducting their investigations at a nearby lab facility.

Following are the quotes from clinicians from different specialties explaining the kind of challenges they faced and how they overcame those challenges.

**Pediatrician:** *“It is particularly challenging to provide teleconsultations for kids since the clinical examination is so important to their care, and telemedicine doesn’t have any options to go beyond inspection, and on top of that, you’ll have to rely on whatever video/image quality you have. Parents usually are not having a good internet connection. Children also require follow-up visits, but despite repeated calls, patients frequently fail to show up.”*

**Surgeon:** *“Due to lack of physical examination, assessment of the surgical site is difficult. I am calling my patients to come to OPD physically as I don’t want to get them infected at surgical sites.”*

**Orthopedic surgeon:** *“Lack of physical examinations is leading to misdiagnosis sometimes. There are also issues with getting detailed patient reports if they used to take treatment from somewhere else. They start showing their report through the video camera during teleconsultation, but that doesn’t work well. I am asking patients to send their reports on WhatsApp so that I can see them properly. There are also language issues sometimes, for which I ask patients to write about their problems and text to me.”*

**Dermatologist 1:** *“I am asking patients to send their reports and photos on my number, as it is not visible properly during teleconsultation. But it breaches the patient’s privacy, and as I use my number, it is getting circulated everywhere. I tried to use email, but most patients are comfortable with WhatsApp only.”*

**Dermatologist 2:** *“Many male patients do not want to show their private parts on video camera even though they have dermatological complaints over there, and even few of them are hesitant to send the images also on WhatsApp.”*

**Nephrologist:** *“I am unable to see the patient’s clinical condition properly through video calling. Besides that, in nephrology, due to frequent investigations, patients have the bulk of reports with them. It is essential to correlate with the previous reports while prescribing the doses of the drugs. I ask patients to send the reports on WhatsApp, but they send them multiple times, so it is tough and time-consuming to see such lengthy reports for each patient on mobile. I somehow manage by convincing patients to be precise while sending the reports.”*

**Cardiothoracic surgeon:** *“Major barrier of telemedicine is not to get the desired investigations of the patients, for example, PT-INR test which is essentially required in valve replacement surgery. Somehow I am trying to manage by asking patients to get investigations done at a nearby lab and report me telephonically.”*

**Radiation Oncologist:** *“Because some patients belong to remote areas, there are network issues, and labs are also unavailable, making it difficult to acquire accurate reports from the patient’s side, and start the treatment.”*

**Pulmonary physician:** *“Many patients are not well versed with digital technology, so they found difficulty in getting appointments and managing online stuff. This e-illiteracy leads to frustration among them, and eventually, they stop responding properly. My team and I are giving a lot of time to counsel them to mitigate this issue.”*

**Gastro Surgeon:** “*Patients don’t pick up the calls on the scheduled time even after multiple attempts of calling. Later on, they often call on odd spells at their convenience, and my team and I are giving them consultations beyond OPD hours.”*

**Neurosurgeon:** *“Neurological examination, including assessment of the status of the patients, is a vital part of diagnosis in our department. It cannot be done through teleconsultations, especially when videos are blurred and patients don’t have good network connectivity. To overcome the problem, I am asking patient attendants to send the recorded videos and photographs of patients after giving them the necessary instructions so that at least I can see the clear images and videos.”*

**Obstetrician:** *“Certain conditions requiring the physician’s attention are missed in the teleconsultations. Sometimes we came to know about that condition in the second or third consultation though patients don’t hide that intentionally, it is missed or overlooked sometimes.”*

**Psychiatrist:** *“I didn’t face any issue while talking with the patients or attendants of the patient during teleconsultation. The problem is that I cannot prescribe many psychotropic medications due to strict guidelines.”*

**Physiotherapist:** *“We demonstrate to the patients to undergo different exercises. These physical exercises need accuracy and precision because if patients do them wrongly, the effects may be deleterious. In such situations, making the patient understand the physiotherapy treatment properly using telemedicine is pretty challenging.*

***Oncologist:*** *Here, the telemedicine setup is excellent with very good network connectivity, and I am relieved that I was able to follow up with my cancer patients.*

***General Medicine:*** *I am not sure how telemedicine is perceived by others. But for sure, it prevents the transmission of infection and saves a lot of time for patients.*

Nearly all of the clinicians acknowledged that they were familiar with the goals of telemedicine and were aware of its benefits and drawbacks. Most doctors believed that telemedicine is convenient for patients (77%) and prevents the transmission of infection (94.2%), although 28.8% of them found it inconvenient to use by themselves. Almost 60% of the clinicians thought that telemedicine saves time, while 26.7% disagreed with this (Table 2).

**Table 2:** Perception of doctors delivering clinical consultations through telemedicine

Variables	No (%)					Med.	Mod.
	SA	A	NS	D	SD		
Know the purpose of telemedicine	43 (82.7)	9 (17.3)	0 (0.0)	0 (0.0)	0 (0.0)	5	5
Understand its advantages & disadvantages	42 (80.8)	9 (17.3)	1 (1.9)	0 (0.0)	0 (0.0)	5	5
Convenient for doctors	2 (3.8)	25 (48.1)	10 (19.2)	10 (19.2)	5 (9.6)	4	4
Convenient for patients	16 (30.8)	24 (46.2)	8 (15.4)	2 (3.8)	2 (3.8)	4	4
Prevents transmission of infection	34 (65.4)	15 (28.8)	1 (1.9)	1 (1.9)	1 (1.9)	5	5
Saves time	18 (34.6)	13 (25.0)	7 (13.5)	13 (25.0)	1 (1.9)	4	5
Patient conditions can be assessed just like in-person visits	2 (3.8)	22 (42.3)	9 (17.3)	11 (21.2)	8 (15.4)	3	4
Patients can be explained their medical conditions just like in-person visits	4 (7.7)	31 (59.6)	6 (11.5)	9 (17.3)	2 (3.8)	4	4
Patients can understand their condition just like in-person visits	2 (3.8)	16 (30.8)	13 (25.0)	16 (30.8)	5 (9.6)	3	2
It can replace part of in-person visits	3 (5.8)	23 (44.2)	11 (21.2)	10 (19.2)	5 (9.6)	3.5	4
Needed in emergent situations such as COVID-19	42 (80.8)	10 (19.2)	0 (0.0)	0 (0.0)	0 (0.0)	5	5
Needed regardless of emergent situations	6 (11.5)	28 (53.8)	9 (17.3)	4 (7.7)	5 (9.6)	4	4
Miscommunication is an issue with it	20 (38.5)	26 (50)	5 (9.6)	0 (0.0)	1 (1.9)	4	4
Medical disputes and conflicts present	2 (3.8)	16 (30.8)	13 (25)	19 (36.5)	2 (3.8)	3	2
Connectivity issues are present	20 (38.5)	25 (48.1)	1 (1.9)	3 (5.8)	3 (5.8)	4	4
Poor quality of investigation reports are found	7 (13.5)	29 (55.8)	12 (23.1)	2 (3.8)	2 (3.8)	4	4
Overall I am satisfied with the telemedicine	1 (1.9)	30 (57.7)	13 (25)	7 (13.5)	1 (1.9)	4	4
I would use telemedicine services again	4 (7.7)	30 (57.7)	14 (26.9)	3 (5.8)	1 (1.9)	4	4

The claim that telemedicine may be used to evaluate patients’ problems in the same way as the in-person visit was disputed by more than half of the clinicians or met with ambiguity on their part. Most doctors agreed (mode = 4) that they can explain a patient’s medical condition to them just like during an in-person visit. Still, most disagreed (mode = 2) that telemedicine enables patients to understand their conditions in a way comparable to in-person consultations. Telemedicine couldn’t even partially replace a patient’s physical visits, according to about a third of doctors. Every clinician confirmed the requirement for telemedicine services in an emergency like COVID-19. However, 17.3% of doctors disapproved of its application in non-emergency situations (Table 2).

Almost all of the doctors mentioned the problem of miscommunication in the telemedicine system. Nearly one-third (34.6%) of the respondents agreed that there were perceived medical disputes and conflicts in the telemedicine system. Most participants (86.6%) stated that telemedicine has issues like time delays and inadequate network connectivity. Poor reports were observed by 69.3% of the clinicians using telemedicine networks. Up to 15% of physicians expressed dissatisfaction with the telemedicine system, and 7.7% said they would never use it again (Table 2).

Further analysis showed that none of the socio-demographic variables (age, sex, and type of occupation) were significantly associated with the perception of clinicians providing telemedicine services (Supplementary Table 1).

Over half of the patients (58.2%) reported that it was their first teleconsultation, while 30.6% had two to five teleconsultations during the year. Nearly 34% of the participants answered that they would have had to drive too far to access health services if telemedicine had not been offered. After teleconsultation, about 87% of patients were asked to the physical OPD for further investigation and clinical evaluation. The majority of the participants (82.8%) stated that their queries were accomplished on WhatsApp by doctors. Almost a third of the participants (35.8%) claimed they spent less than 5 minutes on teleconsultation. The mean time spent in recent teleconsultation services was 4.48 ± 3.12 minutes (Table 3).

**Table 3:** Attributes of Tele-Consultation services received by patients

Variables	No.	%
**Number of Tele-Consultations received**		
Only One	78	58.2
2 to 5	41	30.6
6 to 10	12	9.0
More than 10	3	2.2
**Had to travel too far if telemedicine had not been available**		
Yes (Out of Jodhpur)	46	34.3
No (Within Jodhpur)	88	66.7
**Called to physical OPD for further investigation**		
Yes	117	87.3
No	17	13.7
**Queries accomplished on the WhatsApp**		
Yes	111	82.8
No	23	17.2
**Time spent in Tele-consultation**		
≤ 2 minutes	45	33.6
3 to 4 minutes	3	2.2
5 minutes	74	55.2
> 5 minutes	12	9.0

Patients were asked about the challenges they faced during teleconsultations. Following are some of the quotes from patients:

**Patient 1:** *“Getting timely appointments and follow-up dates arechallenging. Long wait times might be problematic in emergency scenarios. I was not given a date for my next follow-up visit by the doctor.”*

**Patient 2:** *“The doctors did not pick up the calls and didn’t reply. This lack of communication created difficulty in an emergency. To whom should I call in an emergency? There is a dire need to improve this system.”*

**Patient 3:** *“My date for the operation was postponed two times, and still, I don’t know whether I will be operated on or not. I am concerned that my condition should not deteriorate due to this long waiting.”*

**Patient 4:** *“It is excellent service provided by the hospital; I am delighted with the attitude of doctors and the time and treatment they provide. But there is always a scope for improvement.”*

**Patient 5:** *“I faced difficulty in registration and getting a timely appointment. Physical OPD is always better than video conferencing.”*

**Patient 6:** *“Doctor did not reply after receiving investigation reports neither they sent the prescription nor reply for treatment and consultation. I tried to connect to the department phone in the hospital, but nobody picked up.”*

**Patient 7:** *“Doctor did not specify the dosage of medicines and did not answer the queries asked. I got the prescription but am unclear about the doses and frequency. There was abysmal communication. Doctors and patients should talk more freely.”*

The majority of participants agreed or strongly agreed that their experiences were positive in terms of ease of registration (82.1%), audio quality (100%), freedom to discuss medicine (94.8%), and comprehension of the diagnoses or recommendations made (88.1%). However, the majority (82.1%) of patients were equivocal regarding the video quality during teleconsultants. Nearly all the participants (97%) denied feeling uncomfortable in front of the camera. For the majority of respondents (82.1%), the overall telemedicine consultation experience was positive (Table 4).

**Table 4:** Perception of Patients receiving clinical consultations through telemedicine

Variables	No (%)					Med.	Mod.
	SA	A	NS	D	SD		
**Experience**							
Ease of registration	19 (14.2)	91 (67.9)	0 (0.0)	18 (13.4)	6 (4.5)	4	4
The quality of the video was good	2 (1.5)	22 (16.4)	110 (82.1)	0 (0.0)	0 (0.0)	3	3
The quality of the audio was good	14 (10.4)	120 (89.6)	0 (0.0)	0 (0.0)	0 (0.0)	4	4
Able to talk freely about medicine	10 (7.5)	117 (87.3)	6 (4.5)	1 (0.7)	0 (0.0)	4	4
Able to understand the diagnosis and recommendations	13 (9.7)	105 (78.4)	10 (7.5)	4 (3.0)	2 (1.5)	4	4
Feel uncomfortable in front of the camera	0 (0.0)	0 (0.0)	3 (2.2)	131 (97.0)	0 (0.0)	2	2
Overall experience was good	39 (29.1)	71 (53.0)	14 (10.4)	10 (7.5)	0 (0.0)	4	4
**Satisfaction**							
Satisfied with the time spent during teleconsultation by the clinician	36 (26.9)	73 (54.5)	19 (14.2)	3 (2.2)	2 (1.5)	4	4
Satisfied with advice and treatment	67 (50.0)	38 (28.4)	12 (9.0)	12 (9.0)	5 (3.7)	4.5	5
Satisfied with the attitude and communication of doctors	69 (51.5)	36 (26.9)	13 (9.7)	12 (9.0)	4 (3.0)	5	5
**Attitude**							
Telemedicine made healthcare easier during COVID19	84 (62.7)	48 (35.8)	1 (0.7)	1 (0.7)	0 (0.0)	5	5
Willing to participate in another teleconsultation	20 (14.9)	73 (54.5)	36 (26.9)	4 (3.0)	1 (0.7)	4	4

Most of the patients expressed satisfaction with the length of the teleconsultation (81.4%), the advice and care they received (78.4%), and the manner and communication of the clinicians (78.4%). Although all participants had a positive attitude that telemedicine made healthcare easier during the COVID-19 pandemic, five patients refused to engage in another teleconsultation, and 26.9% expressed their uncertainty (Table 4).

On further analysis, none of the socio-demographic variables of the patients was found to be significantly associated with their experience, satisfaction, and attitude towards telemedicine (Supplementary Table 2).

A crystalized synthesis of challenges from the clinician side, patient side, and IT/infrastructure-related issues along with possible solutions for telemedicine implementation from qualitative findings is mentioned in Table 5.

**Table 5:** Synthesis of challenges and possible solutions of telemedicine from qualitative findings

**Challenges for telemedicine**	
**Challenges to the clinicians**	
• Unable to carry out the clinical examinations	
• Lost to follow-ups of the patients due to no response	
• Unable to get the desired investigations	
• Issues with getting detailed old reports if patients used to take treatment from somewhere else	
• Unable to do the inspection properly and see the reports on live camera	
• Difficulty in studying bulk of investigation reports on WhatsApp/mobile	
• Certain conditions requiring the physician’s attention are missed or overlooked	
• Difficulty in prescribing many psychotropic medications due to strict guidelines	
• Patients can not be explained their medical conditions just like in-person visits	
• Challenges in making the patient understand the physiotherapy treatment precisely	
• The hesitancy of the patients to show their private parts on the video camera	
• Breach of privacy due to wider circulation of private mobile numbers among people	
• E-illiteracy of patients	
• Non-response from patients at the scheduled teleconsultation time	
• Call by patients on odd times	
• Miscommunication	
• Medical disputes and conflicts	
**Challenges to the patients**	
• Difficulty in registration and getting a timely appointment	
• Issues in getting follow-up dates	
• Postponement of dates for operations and follow-ups	
• Lack of timely response/communication from department/hospital	
• Non-response from doctors over the phone	
• Lack of communication by doctors after sending investigation reports	
• The doctors do not specify the dosage of medicines and do not answer the queries asked	
• Can not understand their condition just like in-person visits	
**IT/ infrastructure related issues**	
• Poor internet connection on the patient side	
• Poor video/image quality	
• Unavailability of labs in remote areas	
**Possible solutions implemented by the doctors**	
• By calling the patients to OPDs physically	
• By asking patients to send their photos and reports on WhatsApp number	
• By asking patients to send the recorded videos and photographs after giving them the necessary instructions	
• By convincing patients to be precise while sending the reports	
• By asking patients to get investigations done at a nearby lab and report telephonically	
• By giving them consultations beyond OPD hours	
• By giving sufficient time for counselling patients to mitigate their frustration due to e-illiteracy	

## Discussion

The scientific community strongly recommended the implementation of telemedicine even in pre-COVID times to embrace telehealth modalities because of its ease of use, its tendency to improve outcomes and communication, its low-cost implementation, and the benefit of reduced travel time [8]. However, before the COVID-19 pandemic, telemedicine was not widely used in India. This adhoc implementation was new to the country’s entire Health Care Delivery System (HCDS) and the patients. So, this study attempted to explore providers’ and recipients’ experiences and perspectives about telemedicine services.

In the current study, doctors understood telemedicine and its benefits well, and half of the doctors opined that telemedicine could partially replace a patient’s physical visits. According to a study conducted at a comparable apex institution in Delhi, not many doctors were unaware of telemedicine even a decade ago, and almost 60% believed telemedicine would reduce the burden of OPDs [9].

Physicians’ specialized digital communication skills, which are very different from face-to-face interactions, are essential for the successful implementation of telemedicine. Although there has been a significant increase in telemedicine education in medical school from 41 to 60% from 2013 to 2018 in the pre-COVID era [6], these competencies and skills are not imparted through the existing medical curriculum in India [7]. The present study also revealed that almost half of the doctors did not favor telemedicine being convenient for doctors.

Clinicians were found to have a mixed attitude towards telemedicine use. Almost 60% of clinicians thought telemedicine saves time. Still, nearly half of the doctors did not agree that the quality of care delivered through telemedicine is at par with in-person care. These findings are supported by a study conducted on family physicians, which highlighted that nearly half of the doctors thought that telemedicine takes less time than in-person visits, but 70% of them perceived that office visits are more efficient [10]. Numerous previous research indicates that doctors typically had a favorable attitude toward the introduction of telemedicine [11–14].

All the clinicians believed that telemedicine is helpful in COVID-19-like pandemic situations, and nearly two-thirds agreed that it is a valuable technique to provide health services even in non-emergency scenarios. This result is consistent with a survey of Indian doctors, where 71% viewed telemedicine as a possible tool for future healthcare delivery [15]. This finding is also supported by various studies done post-COVID pandemic that agreed on telemedicine as a viable approach for providing medical care to patients [16–18]. Its usefulness during the post-pandemic period has also been evidenced [19]. The reasons for this stimulation may be due to reduced cost, travel, and prompt treatment, along with its usefulness in combating the pressure on the health system for delivering health care with a limited healthcare workforce [20].

In this study, most doctors believed that telemedicine prevents the transmission of infection. This finding is corroborated by published literature where doctors perceived this as one of the crucial reasons for preferring telemedicine over in-person visits [10]. It was found that nearly two third of the doctors were satisfied with the telemedicine system and accepted that they would use it again. These findings are supported by a study by Avik Ray et al. in Madhya Pradesh [21]. The possible reasons may be cutting down the unnecessary footfall in the hospitals and reducing doctor-patient contact in such pandemic times [22].

As far as challenges are concerned, major barriers faced by clinicians during teleconsultation were difficulty in the physical evaluation of patients, miscommunication, network connectivity, medical disputes, and poor quality of investigation reports. Difficulty in reaching the correct diagnosis using teleconsultation due to the lack of physical examination is a major challenge for telemedicine which many authors explored [19, 23]. Miscommunication and poor network connectivity also pose a major challenge in telemedicine systems [18, 24].

In the present study, the majority of patients had positive experiences in terms of ease of registration, audio quality, freedom to discuss medicine, comprehension of the diagnoses or recommendations made by clinicians, length of the teleconsultation, the advice and care they received, and the manner and communication of the clinicians. For the majority of the respondents, the overall telemedicine consultation experience was good. These findings are well supported by published literature depicting patient satisfaction with telemedicine during the COVID-19 pandemic [25–30]. Similar to the study by Nguyen M et al., all the patients in the present study had a positive attitude that telemedicine made healthcare easier during the COVID-19 pandemic [31]. The possible explanation for this positive attitude may be the low cost, and lack of accessibility to hospitals, as the governments recommended people stay indoors during the COVID pandemic. It has already been proved that health services delivered through telemedicine result in lower patient monetary as well as time-related costs [32, 33]. A systematic review of economic evaluations of telemedicine proved its cost-effectiveness in major medical departments [34].

There are many limitations in this study. One of the limitations was the short time frame, which statistically restricted the sample size calculation. An arbitrary number of 2 healthcare workers and 4-5 patients from each department was decided based on the feasibility. The second limitation is that this study is cross-sectional, so we could not establish any causal relationship with specific factors. Third is that there may be a possibility of intruding social desirability bias in the study as a consequence of reporting more desirable attributes both by clinicians as well as patients.

From a policy standpoint, there is a need to work on identified barriers and challenges for the smooth implementation of teleconsultation services and benefit the underprivileged population. Though the sustainability and long-term implementation of telemedicine services require the availability of several elements including governance and political will, organizational support, adequate financing, appropriate technologies, active and targeted communication strategies, collaborative approach, and awareness generation among people to make them e-literate [35]. But this needs to be dealt with as part of a continuous process. The scope of telemedicine services, obstacles and challenges in implementation, and patient satisfaction levels need to be explored further through multi-centric studies.

## Conclusion

Telemedicine became a much-needed tool to provide health services during the COVID-19 pandemic. Though there were some challenges in implementing telemedicine services, like difficulty in a physical evaluation followed by miscommunication and network connectivity issues, the healthcare providers perceived it helpful. Difficulty in registration, lack of communication, and a deep-rooted mindset of physical consultations were the primary concerns from the patient side. The majority of the patients were satisfied with teleconsultation services. Telemedicine was found to be a viable platform for healthcare workers and ass well as patient-side, so it can be scaled up as an essential tool to provide health services across the nation.

## Supplementary Information

**Additional file 1 Supplementary Table 1.** Association of perception of doctors regarding delivering telemedicine services with socio-demographic variables. **Supplementary Table 2.** Association of perception of the patient regarding receiving telemedicine services with socio-demographic variables.

## Data Availability

All data generated or analysed during this study are included in this published article [and its supplementary information files].

## Abbreviations

A: Agree
D: Disagree
GoI: Government of India
HCDS: Health Care Delivery System
MoH&FW: Ministry of Health and Family Welfare
NS: Not Sure
OPDs: Outpatient Departments
RMPs: Registered Medical Practitioners
SA: Strongly Agree
SD: Strongly Disagree
SRs: Senior Residents
