# Supplementary material for: Is telemedicine a holy grail in healthcare policy: clinicians’ and patients’ perspectives from an Apex Institution in Western India
Source: BMC Health Serv Res. 2023 Feb 15;23:161. doi: 10.1186/s12913-022-09013-y (PMC9930698; doi:10.1186/s12913-022-09013-y)
Supplement: Supplementary file 1 — Additional file 1 Supplementary Table 1. Association of perception of doctors regarding delivering telemedicine services with socio-demographic variables. Supplementary Table 2. Association of perception of the patient regarding receiving telemedicine services with socio-demographic variables. [file 12913_2022_9013_MOESM1_ESM.docx]

**Supplementary Table 1: Association of perception of doctors regarding delivering telemedicine services with socio-demographic variables**

| **Variables** | **Mean Rank** | | | | | | | | | |
| --- | --- | --- | --- | --- | --- | --- | --- | --- | --- | --- |
|  | **Age (yrs.)** | | | | **Gender** | | | **Occupation** | | |
|  | **<30** | **30-40** | **>40** | **^*^Sig.** | **M** | **F** | **^#^Sig.** | **Faculty** | **SR** | **^#^Sig.** |
| Know the purpose of telemedicine | 26 | 26.9 | 25 | 0.88 | 27 | 26 | 0.93 | 25.8 | 27 | 0.69 |
| Understand its advantages & disadvantages | 25 | 27.5 | 24 | 0.66 | 26 | 29 | 0.33 | 26.2 | 27 | 0.85 |
| Convenient for patients | 26 | 27.6 | 19 | 0.48 | 27 | 26 | 0.98 | 23.9 | 28 | 0.29 |
| Convenient for doctors | 25 | 28.5 | 16 | 0.2 | 26 | 28 | 0.78 | 25.5 | 27 | 0.68 |
| Prevents transmission of infection | 32 | 24.3 | 21 | 0.07 | 26 | 29 | 0.49 | 24.8 | 28 | 0.43 |
| Saves time | 31 | 25.2 | 18 | 0.17 | 27 | 25 | 0.69 | 22.5 | 29 | 0.12 |
| Patient conditions can be assessed just like in-person visits | 32 | 23.9 | 24 | 0.14 | 25 | 31 | 0.28 | 22.2 | 29 | 0.09 |
| Patients can be explained about his/her medical conditions just like in-person visits | 30 | 24.5 | 30 | 0.41 | 27 | 24 | 0.56 | 25.8 | 27 | 0.77 |
| Patients can understand their condition just like in-person visits | 34 | 22.6 | 28 | 0.04 | 27 | 26 | 0.84 | 22 | 29 | 0.08 |
| It can replace part of in-person visits | 28 | 24.5 | 38 | 0.2 | 26 | 30 | 0.34 | 28.2 | 25 | 0.49 |
| Needed in emergent situations such as COVID19 | 30 | 25.8 | 19 | 0.12 | 26 | 29 | 0.34 | 25 | 27 | 0.41 |
| Needed regardless of emergent situations | 22 | 27.8 | 34 | 0.23 | 27 | 25 | 0.71 | 29.5 | 25 | 0.21 |
| Miscommunication is an issue with it | 31 | 24.7 | 23 | 0.28 | 27 | 24 | 0.56 | 21.4 | 30 | 0.03 |
| Medical disputes and conflicts present | 26 | 26.1 | 30 | 0.89 | 25 | 32 | 0.14 | 30.9 | 24 | 0.08 |
| Connectivity issues are present | 29 | 25.7 | 21 | 0.5 | 26 | 27 | 0.89 | 21.3 | 30 | 0.03 |
| Poor quality of investigation reports are found | 32 | 25.2 | 16 | 0.07 | 26 | 27 | 0.93 | 20.2 | 31 | 0.01 |
| Overall I am satisfied with the telemedicine | 28 | 25.6 | 29 | 0.82 | 27 | 27 | 1 | 23.8 | 28 | 0.26 |
| I would use telemedicine services again | 24 | 27.7 | 28 | 0.62 | 27 | 23 | 0.38 | 27.5 | 26 | 0.67 |

^*^Kruskal-Wallis H, ^#^Mann-Whitney U

**Supplementary Table 2: Association of perception of the patient regarding receiving telemedicine services with socio-demographic variables**

| **Variables** | **Mean Rank** | | | | | | | | | | | | |
| --- | --- | --- | --- | --- | --- | --- | --- | --- | --- | --- | --- | --- | --- |
|  | **Age (yrs)** | | | | **Gender** | | | **Education (Std.)** | | | **Occupation** | | |
|  | **≤14** | **15-59** | **≥60** | **^*^Sig.** | **M** | **F** | **^#^Sig.** | **<10th** | **≥10th** | **^#^Sig.** | **^$^E** | **^$^U** | **^#^Sig.** |
| **Experience** | | | | | | | | | | | | | |
| Ease of registration | 57.4 | 67.5 | 85.7 | 0.170 | 68.9 | 65.5 | 0.548 | 58.0 | 60.3 | 0.678 | 64.3 | 57.8 | 0.228 |
| The quality of the video was good | 80.9 | 65.4 | 76.1 | 0.083 | 68.5 | 66.1 | 0.597 | 57.1 | 60.8 | 0.380 | 60.8 | 60.3 | 0.893 |
| The quality of the audio was good | 60.5 | 67.6 | 79.6 | 0.139 | 68.2 | 66.5 | 0.627 | 55.3 | 61.8 | 0.077 | 64.5 | 57.7 | 0.059 |
| Able to talk freely about medicine | 66.0 | 66.6 | 84.1 | 0.130 | 66.1 | 69.5 | 0.388 | 58.1 | 60.3 | 0.587 | 59.1 | 61.5 | 0.543 |
| Able to understand the diagnosis and recommendations | 55.2 | 68.8 | 68.4 | 0.247 | 65.1 | 70.9 | 0.237 | 57.8 | 60.5 | 0.563 | 55.1 | 64.3 | 0.048 |
| Feel uncomfortable in front of the camera | 66.0 | 67.8 | 66.0 | 0.766 | 66.9 | 68.4 | 0.379 | 59.4 | 59.6 | 0.934 | 59.0 | 61.5 | 0.147 |
| Overall experience was good | 69.4 | 67.7 | 60.1 | 0.840 | 69.4 | 64.9 | 0.464 | 55.0 | 62.0 | 0.251 | 60.8 | 60.3 | 0.926 |
| **Satisfaction** | | | | | | | | | | | | | |
| Satisfied with the time spent during teleconsultation by the clinician | 63.5 | 67.8 | 69.3 | 0.907 | 66.7 | 68.6 | 0.761 | 59.1 | 59.7 | 0.926 | 59.5 | 61.2 | 0.772 |
| Satisfied with advice and treatment | 69.2 | 67.0 | 72.0 | 0.924 | 69.8 | 64.3 | 0.376 | 56.0 | 61.4 | 0.373 | 58.7 | 61.7 | 0.615 |
| Satisfied with the attitude and  communication of doctors | 73.2 | 67.3 | 60.1 | 0.726 | 68.7 | 65.9 | 0.651 | 58.1 | 60.3 | 0.724 | 60.3 | 60.6 | 0.954 |
| **Attitude** | | | | | | | | | | | | | |
| Telemedicine made healthcare  easier during COVID19 | 62.0 | 69.1 | 51.1 | 0.301 | 69.3 | 65.0 | 0.445 | 58.4 | 60.1 | 0.756 | 60.4 | 60.6 | 0.967 |
| Willing to participate in another  teleconsultation | 72.6 | 66.9 | 67.9 | 0.857 | 68.6 | 65.9 | 0.662 | 56.9 | 60.9 | 0.500 | 58.5 | 61.9 | 0.564 |

^*^Kruskal-Wallis H, ^#^Mann-Whitney U, ^$^E-Employed, ^$^U-Unemployed
